# The challenges of medically complex breastfed children and their families: A systematic review

**DOI:** 10.1111/mcn.13182

**Published:** 2021-05-06

**Authors:** Lyndsey Hookway, Jan Lewis, Amy Brown

**Affiliations:** ^1^ Department of Public Health, Policy and Social Sciences, College of Human and Health Sciences Swansea University Swansea UK

**Keywords:** breastfeeding, healthcare staff, lactation support, medically complex infant, paediatric care, PICU

## Abstract

Exclusive breastfeeding for the first 6 months and then alongside solid food for the first 2 years and beyond is the gold standard in young child nutrition. There is an abundance of literature relating to the preventative nature of breastmilk and breastfeeding against many infectious diseases and chronic conditions. However, despite medically complex infants and children being a group that could benefit most from continued breastfeeding, breastfeeding duration and exclusivity are lower among more complex paediatric populations. The reasons for this are not well known, and there is a paucity of data relating to supporting infants who have acute or chronic illness, disability or congenital anomaly to breastfeed. This systematic review aimed to understand the challenges of breast/chestfeeding the medically complex child and to establish the gaps in healthcare provision that act as barriers to optimal infant and young child feeding. The search was limited to studies published in English, focused on breastfed sick infants in hospital, with no date limits as there is no previous systematic review. Of 786 papers retrieved, 11 studies were included for review, and seven themes identified. Themes included practical and psychological challenges of continuing to breastfeed in a hospital setting, complications of the condition making breastfeeding difficult, lack of specialist breastfeeding support from hospital staff and a lack of availability of specialist equipment to support complex breastfeeding. The findings affirm the lack of consistent high‐quality care for lactation support in paediatric settings and reinforce the need for further focused research in this area.

**Key messages**

- There are limited data relating to breastfeeding medically complex children. Existing literature focuses on specific infant conditions. Support to facilitate breastfeeding is generally considered suboptimal.
- Breastfeeding medically complex infants is more challenging. There are practical and psychological parent challenges, as well as infant‐related difficulties. There are also shortcomings with lactation support within paediatrics, a lack of training for healthcare professionals and challenges with provision of and education in using specialized infant feeding equipment.
- Parents of medically complex breastfed children have unique needs, which need to be addressed with research, policy and training in order to optimize outcomes for this population.

## 1 INTRODUCTION

Breastfeeding is the biologically normal way to feed infants and children up to the age of 2 years and beyond (World Health Organization (WHO), 2018). Not breastfeeding is associated with a greater risk of adverse health issues for infants including infectious disease, obesity and diabetes and a rise in reproductive cancers, heart disease and diabetes for women (Victora et al., 2016). Not meeting breastfeeding goals is also associated with higher rates of maternal depression and grief (Borra et al., 2015; Brown, 2016; Fahlquist, 2016). Breastfeeding is perhaps even more important for medically complex children, due to the immunologic protection it confers; for energy consumption when solid foods may be difficult to digest; and for comfort through illnesses and procedures (Edwards & Spatz, 2010; Spatz, 2012; Thomas, 2020). Breastfeeding is also known to be effective in managing procedural pain, though it has so far mainly been studied in the context of vaccination (Harrison et al., 2016). Breastfeeding is a way of parenting and is often used as a tool to calm and soothe a young child; it cannot be distilled down to caloric intake alone (Brown, 2018; Brown et al., 2018).

However, evidence from studies around the world suggests that breastfeeding duration and exclusivity is lower and more difficult to achieve among medically complex children for a variety of reasons. For example, one UK literature review found that children with Down syndrome have lower rates of breastfeeding and those who managed to breastfeed only could because they had more support (Sooben, 2012). This was echoed in a Puerto Rican study (Colón et al., 2009). A recent case report compared volumes ingested by an infant with Down syndrome with an infant without Down syndrome and found that low intra‐oral pressure, large tongue and less effective suckling were clinically significant (Coentro et al., 2020). Meanwhile, Torowicz et al. (2015) studied infants with a congenital heart defect, noting that the high‐stress environment makes establishing a milk supply more challenging. Finally, Rivera et al. (2008) explored the complexities of breastfeeding infants with spina bifida and concluded infant instability after surgery was not the biggest barrier—rather, it was the clinical environment, lack of medical staff knowledge and hospital routines.

It is important to understand how and why breastfeeding is more challenging for parents of medically complex children in order to target services and support to enable them to meet their feeding goals. Understanding the challenges could also lead to more specific training for healthcare staff so that they are able to support families more effectively and skilfully. Currently, there is little formal guidance for breastfeeding medically complex children. Although the policies of the Baby Friendly Hospital Initiative (BFHI) and the Baby Friendly Initiative (BFI) UK promote, protect and support breastfeeding, their policies do not cover paediatrics where medically complex children will be cared for. Whereas children who are diagnosed antenatally with a congenital anomaly will be cared for in the neonatal unit, where staff are likely to have been trained in how to support breastfeeding and maintain milk supply, children cared for in the cardiac intensive care unit (CICU), paediatric intensive care unit (PICU), emergency department or general medical or surgical paediatric ward may be cared for by staff with very little breastfeeding training. Furthermore, infants who start off in the neonatal unit may be transferred to one of these paediatric settings, meaning that their experience of breastfeeding support may change.

Despite the known difficulties of feeding medically complex children (Coates & Riordan, 1992), there is no systematic review of their breastfeeding experience within the paediatric setting. A synthesis of studies exploring the experiences of parents breastfeeding their medically complex children may illuminate areas for prioritization of training and support. The purpose of this systematic review is therefore:

1. To establish the existing body of knowledge around the challenges and needs of parents breastfeeding their sick infants or children in the paediatric setting.
2. To identify gaps in healthcare provision that act as barriers to maintaining breastmilk supply and facilitating breastfeeding in the medically complex paediatric population.

## 2 METHODOLOGY

To address the research questions, the search strategy (Table 1) and eligibility criteria (Table 2) were designed in line with the PICOS criteria (population, intervention, comparator, outcomes, study design or setting). This is a modification of the PICO criteria, which omits study design or setting and is more commonly used to search for quantitative studies (Methley et al., 2014). Compared with the PICO criteria, PICOS, with the added component of study design and setting, is useful when time or resources are limited and is also more favourable when studies are generally qualitative (Cooke et al., 2012; Methley et al., 2014).

**TABLE 1:** Keywords used in article search

Search number	PICOS component	Search terms (BOOLEAN operator OR)
1	Population	Child, children, babies, baby, infant
2	Intervention	Sick, disease, illness, disability, congenital anomaly, cleft lip, childhood cancer, syndrome, sick children, child with disability
N/A	Comparator	N/A
3	Outcomes	Breastfeeding, breast feeding, breastmilk, human milk, EBM
4	Study design and setting	(Any design, any country) hospital, PICU, paediatrics, paediatrics

**TABLE 2:** Inclusion and exclusion criteria

Inclusion criteria	Study written in English
	Original research article
	Mothers or parents who were directly breastfeeding or providing breastmilk by expressing and bottle feeding, whether exclusive or partial
	Paediatric ward or PICU setting
	Studies that included staff views and training on breastfeeding as well as parent experience
	Studies exploring parental challenges of breastfeeding
	Studies exploring challenges of initiating, maintaining or increasing milk production for sick children
	Studies exploring the challenges of providing breastmilk via alternative feeding routes, such as nasogastric tube or gastrostomy tube
Exclusion criteria	Written in another language
	Exclusively formula‐fed children and formula‐feeding parents
	Studies focused on another aspect of child feeding, for example, solid food
	Community setting
	NICU setting
	Maternity unit or transitional care
	Described a well child with a parent with disability or illness
	Described reasons for cessation of breastfeeding that were not related to child illness
	Compared health outcomes between children who were and were not breastfed in infancy
	Described breastfeeding as a health promotion or preventative strategy
	Described practical or theoretical feasibility of breastfeeding among children with disability
	Expert opinion or theoretical recommendations regarding feasibility without any reference to parental views
	Discussed strategies to increase breastfeeding rates in the general population

### 2.1 Eligibility criteria

Both published and unpublished studies using any methodology were eligible if they met the inclusion criteria (see Table 2). Literature was included from anywhere in the world, as there may be examples of good practice, as well as higher breastfeeding rates in resource‐poor as well as resource‐rich countries (Victora et al., 2016). No date limits were set as there is no previous systematic review, although where located studies were dated, their results were treated with caution.

All studies whose focused population was breastfed children with acute or chronic illness, disability or congenital anomaly were considered. An acute illness is experienced by a child who is usually healthy but experiences a brief illness requiring medical treatment, such as an acute infection, sepsis or accident such as a burn or injury. A chronic illness is a condition that requires observation, monitoring or medical intervention and treatment for many weeks, months or years, such as diabetes, asthma, epilepsy or cancer. Chronic conditions may also sometimes require surgery. A disability is a condition that is often diagnosed antenatally or soon after birth, affecting physical, intellectual or communication abilities, and includes conditions such as Down syndrome, cerebral palsy or Prader–Willi syndrome. A congenital anomaly is a structural or functional anomaly that occurs during intrauterine life that may or may not have serious medical consequences. Many congenital anomalies will require medical or surgical intervention, such as cardiac defects, cleft lip or palate, spina bifida or congenital diaphragmatic hernia.

The neonatal intensive care unit (NICU) as a setting was excluded because Baby Friendly standards already apply to this area, and many staff working within the NICU have already received training in supporting breastfeeding and lactation. Combining data from both the NICU and paediatric settings would therefore potentially confuse the data, as parents in different areas may have different experiences of care.

Publication or researcher bias is a significant problem in academic literature and refers to the tendency to favour publication of statistically significant and positive findings, leaving the null results unpublished. This can unfairly skew the data available and lead to exaggerated emphasis in some subjects (Van Aert et al., 2019). Every effort was made to both avoid and account for publication bias through not excluding studies with non‐statistically significant results, including unpublished data, small studies and studies conducted in many counties, including low‐ and middle‐income countries (Ekmekci, 2017).

It is best practice to have two reviewers to search and sift the literature to ensure inter‐rater reliability; however, this was not possible, as this work forms part of a thesis, which Siddaway et al. (2019) point out is a common issue, requiring flexibility. However, inclusion and exclusion criteria were discussed with a second reviewer, reducing the risk of bias.

### 2.2 Search strategy

Literature was sought in January to February 2020 using CINAHL, PubMed, Google Scholar and iFind. Boolean operators (Table 1) were used to blend the keywords, and alternative spellings were used to capture variants of keywords. The literature search yielded 757 studies, dissertations, reports and narrative reviews. In addition, the reference lists of pertinent books and articles were scrutinized to identify further papers that may have been missed.

All retrieved article titles were read initially. Many studies, while including some relevant search terms in the title, were clearly focused on the NICU environment or on breastfeeding being a preventative intervention against illness. Any article that could not be obviously excluded was kept for further investigation.

**FIGURE 1:**
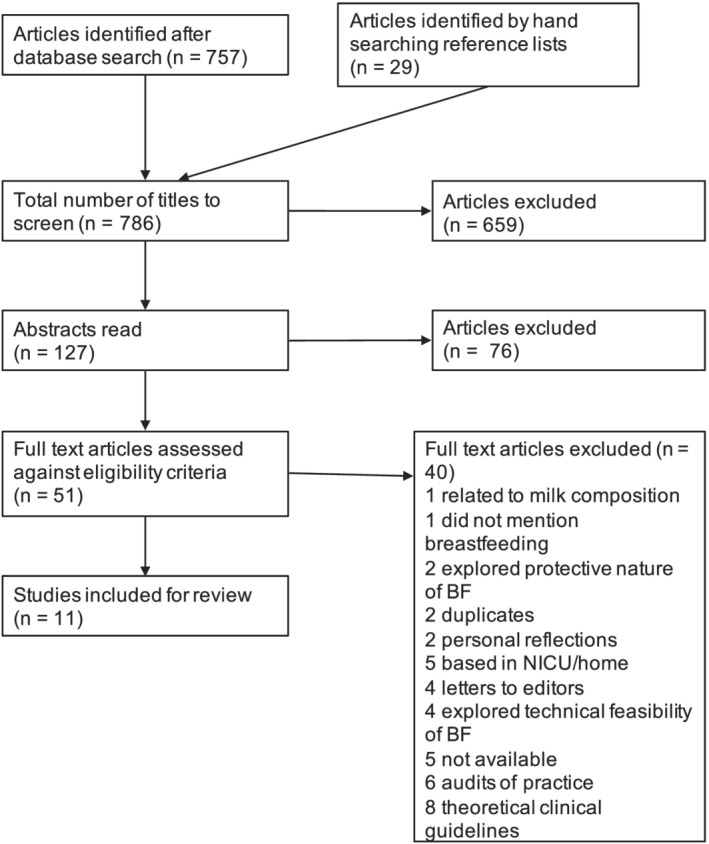
PRISMA flow diagram demonstrating article screening process

After the initial exclusion of articles that did not meet the inclusion criteria, there remained 127 article abstracts to read. Reasons for exclusion are noted in Figure 1. All articles that could not be conclusively accepted or rejected after reading the abstract were kept. After applying the inclusion criteria to the full texts of the remaining papers, there remained 11 articles for review (see Figure 1). A narrative synthesis and thematic analysis were then conducted with the eligible studies.

## 3 RESULTS

A table was created to summarize the main study characteristics and findings to assist with analysis of the remaining 11 papers (Appendix A1). For quality appraisal of the included studies, existing published Critical Appraisal Checklists—including the checklist for qualitative studies and the cohort study checklist (Critical Appraisal Skills Programme UK (CASP UK), 2018)—were used, and for the cross‐sectional studies, an adapted version of the CASP cohort study checklist was designed (Appendix A2). Eight of the studies were qualitative (Table 3). Two studies were cross sectional in design, and there was one cohort study (Table 4).

**TABLE 3:** Quality appraisal summary of eight included qualitative studies

CASP criteria	Met the criteria? (yes, cannot tell, no)							
	Banta‐Wright et al., 2015	Barbas & Kelleher, 2004	Barros da Silva et al., 2019	Moe et al., 1998	Duhn & Burke, 1998	Lambert & Watters, 1998	Lewis & Kritzinger, 2004	Ryan et al., 2013
Q1: Was there a clear statement of the aims of the research?	Y	Y	Y	Y	Y	Y	Y	Y
Q2: Is a qualitative methodology appropriate?	Y	Y	Y	Y	Y	Y	Y	Y
Q3: Was the research design appropriate to the aims of the research?	Y	?	Y	Y	Y	?	Y	Y
Q4: Was the recruitment strategy appropriate to the aims of the research?	Y	Y	Y	Y	Y	Y	Y	Y
Q5: Were the data collected in a way that addressed the research issue?	Y	Y	Y	?	Y	Y	Y	Y
Q6: Has the relationship between researcher and participants been adequately addressed?	Y	N	?	?	Y	N	N	Y
Q7: Have ethical issues been taken into consideration?	Y	N	Y	N	Y	N	N	N
Q8: Was the data analysis sufficiently rigorous?	Y	?	Y	N	Y	?	Y	Y
Q9: Is there a clear statement of findings?	Y	Y	Y	Y	Y	Y	Y	Y
Q10: How valuable is the research?	Y	Y	Y	Y	Y	Y	Y	Y

**TABLE 4:** Quality appraisal summary of two cross‐sectional studies and one cohort study

CASP criteria	Met the criteria? (yes, cannot tell, no)		
	Heilbronner et al., 2017	Madhoun et al., 2019	Rendón‐Macías et al., 2002
Q1: Did the study address a clearly focused issue?	Y	Y	Y
Q2: Was the sample recruited in an acceptable way?	Y	Y	Y
Q3: Was the exposure accurately measured to minimize bias?	N/A	N/A	Y
Q3a: Was the outcome accurately measured to minimize bias?	?	? Possible response bias. Limited SE and ethnic diversity	Y
Q4a: Have the authors identified all important confounding factors?	Half the sample did not have socio‐economic background recorded	? No mention of antenatal education	Y
Q4b: Have the authors taken account of confounding factors in their design and analysis?	?	?	Y
Q5: Was the follow‐up of subjects complete enough?	Y	Y	Y
Q5b: Was the follow‐up of subjects long enough?	N/A	N/A	Y
Q6: What are the results of this study?	Hospitalization with bronchiolitis is negatively associated with duration and exclusivity of breastfeeding, especially in younger infants, and those who were tube‐fed	Cleft lip/palate adversely affects BF duration, exclusivity and experience; however, there was a problem with question wording—did not differentiate between exclusive and partial BF. The parents in this sample were well supported by IBCLCs in the hospital setting and achieved a high rate of provision of breastmilk through pumping, and the sample was also biased towards higher SE status	Infants with congenital malformations are less likely to BF. Mothers cited many reasons, including medical advice, separation and infant disease—especially GI disease
Q7: How precise are the results?	N/A	N/A	? Wide confidence intervals
Q7a: Do you believe the results?	Some absent data	Y	? Limitations not discussed
Q8: Can the results be applied to other populations?	?	? Limited SE variation	?
Q9: Do the results of this study fit with other available evidence?	Y	Y	Y
Q10: What are the implications of this study for practice?	Even short hospital durations may pose a threat to breastfeeding outcome, necessitating more support for breastfed dyads in hospital	Infants with cleft are more likely to struggle with breastfeeding, particularly when the palate is involved	Combined with other research relating to congenital malformations, this study adds to the weight of recommendation for targeted support and multidisciplinary collaboration

Overall, there were eight qualitative and three mixed‐methods studies representing a total sample size of 599 (range: *n* = 5–194). All the studies explored the impact on breastfeeding of illness, disability or congenital anomaly. There was a small clustering of studies in 1998, and of 11 included studies, six were conducted in the United States or Canada. There was just one very small UK‐based study, and it was notable how few illnesses or conditions were explored.

### 3.1 Narrative synthesis

#### 3.1.1 Study quality

The studies all had clearly defined aims and recruitment strategies. All of them explored the impact of various medical conditions on breastfeeding outcomes. The studies addressed and commented on a range of potential confounding factors, including socio‐economic status (SES), degree of infant illness or disability and infant age. Only four studies commented on prenatal intention to breastfeed (Lambert & Watters, 1998; Madhoun et al., 2019; Moe et al., 1998; Rendón‐Macías et al., 2002), which could be significant, as parental motivation is known to be a factor in breastfeeding duration and exclusivity (Claesson et al., 2019).

Only two studies commented on whether the hospital facility was baby friendly accredited (Heilbronner et al., 2017; Rendón‐Macías et al., 2002), which may be relevant because BFI/BFHI status is known to positively influence the initiation of breastfeeding, though there are limited data on the correlation between BFI accreditation and maintenance of breastfeeding (Pérez‐Escamilla et al., 2016), and Baby Friendly standards do not at the present time apply to the paediatric setting.

There were many problems with study quality. Only four studies commented on ethical approval; several studies had missing data and small sample sizes. Most of the studies only accounted for a limited number of confounding variables, and some of the terms were not explicitly defined—such as what constitutes an ‘expert’ (see Table 5).

**TABLE 5:** Summary of strengths and limitations of studies

Study	Strengths	Limitations	Country	Sample size	Year of publication	Design	Population
Banta‐Wright et al. (2015)	Well‐designed study meeting all CASP (2018) checklist criteria Consideration given for the wider impacts of breastfeeding beyond nutrition Good example of how breastfeeding could work even in a condition that is ever changing	Highly motivated sample of mothers with previous experience of breastfeeding, and access to IBCLC may reduce generalizability Small sample (*n* = 10)	USA/Canada	10	2015	Qualitative	Mothers of infants with phenylketonuria (PKU)
Barbas and Kelleher (2004)	All infants had a major cardiac anomaly requiring surgery Parents received education about how to pump, and detail is provided about this teaching Detail is provided about how exclusivity of breastfeeding changed over time and why	Self‐selected sample; may have attracted more motivated parents Mostly high SES and educational status Half the sample had previous breastfeeding experience Conflicting remarks about what helped, with no clarification provided	USA	68	2004	Qualitative	Mothers of infants with congenital heart disease (CHD)
Barros da Silva et al. (2019)	In‐depth data collection capturing the meaning of breastfeeding as a parenting activity to parents A range of feeding experiences were represented	Small sample Confounding variables not commented on Did not comment on additional needs sometimes coexisting with Down syndrome, so hard to tell if feeding difficulties were due to Down syndrome, or a co‐morbidity Possible recall bias due to the ages of children at the time of interview	Portugal	10	2019	Qualitative	Mothers of children with Down syndrome
Duhn and Burke (1998)	Well‐designed study meeting all CASP (2018) checklist criteria	Small sample size In this sample, all the mothers had stopped breastfeeding by 4–6 weeks, so limited long‐term data	Canada	7	1998	Qualitative	Mothers of children with CHD
Heilbronner et al. (2017)	Focused on acute illness; provides an important comparison with chronic conditions. Relatively larger sample Separated infant critical illness from environmental factors as a reason for cessation of breastfeeding	Missing demographic data The study commented that there were difficulties staying in hospital overnight, but no clarification No definition of ‘expertise’ despite the study commenting that the ‘experts’ were not helpful Sample may have been too under‐powered to detect an association between PICU admission and reduction in exclusivity of breastfeeding No detail about the extent of breastfeeding modification, despite this being the study focus	France	84	2017	Cross sectional	Mothers of children with bronchiolitis
Lambert and Watters (1998)	Relatively severe CHD in this study, and most infants were breastfeeding at the time of surgery Parents mostly had breastfed another child, so could separate ‘normal’ breastfeeding challenges from CHD‐related challenges	Small sample Most parents had prior breastfeeding experience Limited SES, educational variability Sample recruited via magazine advert, and the infants represented in the sample were born between 1978 and 1993; potential for recall bias, as well as policy and practice change. Therefore, results should be treated with caution	Canada	12	1998	Qualitative	Mothers of children with CHD
Lewis and Kritzinger (2004)	This study separated the co‐morbidities associated with Down syndrome—such as cardiac defect, low tone and poor suck Explored specific mechanical, growth and emotional challenges Authors explored direct breastfeeding, bottle feeding and tube feeding breastmilk	It is unclear whether the support provided was helpful, as not assessed No clear lactation support programme documented Study conducted in South Africa where services were noted to be insufficient. It is unclear whether the difficulties experienced were due to inadequate support or the medical complexity of the infants	South Africa	20	2004	Qualitative	Mothers of children with Down syndrome
Madhoun et al. (2019)	Large and relatively wide SES variation, including 28% of participants involved in the WIC (women, infants and children) programme Rates of breastfeeding similar to national population statistics Explored both breastfeeding and expressing and separated barriers to breastfeeding from expressing Explored the nuances and impact of different severity of cleft	No differentiation between exclusive and partial breastfeeding Self‐selected sample, so response bias is a risk Did not separate exclusive breastfeeding from any breastfeeding—it is unclear whether those with more breastfeeding challenges were also those with lower exclusivity No discussion about how much milk infants received via tube or whether this was predominantly breastmilk or formula	USA	69	2019	Cross sectional	Mothers of infants with cleft lip/palate
Moe et al. (1998)	Large sample Initiation of breastfeeding matched national statistics at the time of the study The condition shares many features of other conditions that may increase the generalizability (with caution)	Very rare condition that may not be generalizable to a broader population Barriers to breastfeeding not formally assessed but were added as free text comments by parents—therefore, the data captured are not standardized. Some challenges may have been missed	USA/Canada	194	1998	Qualitative	Parents of children with Rubinstein–Taybi syndrome
Rendón‐Macías et al. (2002)	Considers a variety of congenital anomalies Sample closely followed up for 6 months; limits recall bias Reasons for cessation of breastfeeding clearly documented and separated by the infant's condition to explore the impact of different conditions on breastfeeding outcome	May not be generalizable to a wider population due to lack of facilities Older study—cohort was 1997–1998. Study limitations not discussed Combined exclusive and partial breastfeeding Unclear what extent the child's condition had on feeding outcomes as the most significant factor was separation of mothers and infants	Mexico	120	2002	Cohort study	Mothers of infants with congenital abnormalities
Ryan et al. (2013)	Well‐designed study meeting all but one of the CASP (2018) checklist criteria Explored emotional adjustment and coping mechanisms as well as practical barriers and challenges Multiple problems and modes of feeding experienced	Small sample size (*n* = 5) Comments about overusing nasogastric tube causing stress, but there is no context. Unclear whether this was inappropriate tube use or the necessity of the tube itself that caused stress	UK	5	2013	Qualitative	Mothers of children with Down syndrome, cleft and CHD

#### 3.1.2 Study themes

Despite the variable study quality, all included studies contributed to the development of themes because there is so little literature available and all the experiences of the study participants are arguably valid and meaningful. Themes were developed by reading and rereading the papers to become familiarized with their purpose, methods and results.

A theoretical thematic analysis was chosen, because the themes were not necessarily linked to the data being collected in the study in question, although they were explicit within the meaning of the papers. Rather, the data collected were analysed in relation to this research question (Braun & Clarke, 2006).

Seven themes emerged from the literature: parental factors, both practical and psychological; infant factors, relating to both infant illness and instability, as well as how a chronic condition affected their ability to effectively breastfeed; the availability of specialist lactation support; support and information from healthcare professionals; and specialized equipment and resources needed. Eight studies mentioned at least six out of the seven themes and the most prevalent theme related to the inadequacy of healthcare professional support—this was identified as a barrier in every study (see Table 6).

**TABLE 6:** Study themes

Study	Sub‐themes	Themes analysed from data
Banta‐Wright et al. (2015)	Breastfeeding was hard work but important. Mothers had to adapt to infant condition constantly. IBCLCs were hard to find, and healthcare professionals lacked information. Pumps were essential	1, 2, 4, 5, 6, 7
Barbas and Kelleher (2004)	Breastfeeding increased self‐efficacy. Infants were sometimes too sick and too sleepy to feed. IBCLCs were found to be helpful but few of them. Nurses were less helpful. Pumps were essential	2, 3, 4, 5, 6, 7
Barros da Silva et al. (2019)	Practical issues of discomfort and difficult to maintain supply. Breastfeeding was hard, but parents motivated to persevere. Infants often too sick to feed, then once better, very sleepy. Very little support available. Pumps essential and not always available	1, 2, 3, 4, 5, 6, 7
Duhn and Burke (1998)	Parents were exhausted and stressed. Surgery and ventilation were barriers to success. As infants got better, they continued to be sleepy and struggled with weight. Healthcare professionals often used negative language. Lack of support to use equipment though it was essential.	2, 3, 4, 6, 7
Heilbronner et al. (2017)	Parents struggled with practicalities of admission to hospital. Illness severity not linked to breastfeeding rates in this sample. Inadequate lactation support. Poor advice from healthcare professionals. Not enough pumps	1, 3, 5, 6, 7
Lambert and Watters (1998)	Practical milk supply issues. Parents struggled with fatigue and anxiety. Preoperative fasting and surgery was a barrier. Adaptations for sleepy, slow gaining babies needed. IBCLCs were helpful but not enough of them. Healthcare professionals had little training and were not found to be helpful by parents. Lack of privacy and inconsistent advice about equipment noted.	1, 2, 3, 4, 5, 6, 7
Lewis and Kritzinger (2004)	Parents experienced a range of emotions. Infants were often complex and unstable. Infants often had low tone, and adaptations were needed. No IBCLCs, but peer support was helpful. No critique of healthcare professional input. Parents variously used nasogastric and gastrostomy tubes but no information about how these were managed by parents	2, 3, 4, 5, 6, 7
Madhoun et al. (2019)	Milk supply struggles. Anxiety and depression were common. Many breastfeeding problems and lower duration of feeding with cleft palate. IBCLCs accessible but not part of cleft team. Healthcare professionals lacked knowledge and skills. Multiple types of equipment needed	1, 2, 4, 5, 6, 7
Moe et al. (1998)	Feeding challenges were common. Infant supplementation common. Breastfeeding was seen as a positive intervention. Many complex swallowing problems cited, plus low tone and sleepy infants. Lack of lactation support. Healthcare professionals were unsupportive. Specialized techniques were needed	2, 3, 4, 5, 6, 7
Rendón‐Macías et al. (2002)	Perceived low milk supply was common. Fasting protocols and surgery were barriers, and as infants got better, weight problems and poor suck were problematic. Advice to stop breastfeeding was common	1, 3, 4, 6
Ryan et al. (2013)	Perceived low milk supply was common. Parents experienced stress and anxiety frequently. Needing to know fluid volumes was a barrier. Parents were critical of healthcare professionals' support of lactation but felt conflicted as they needed those professionals to care for their infants clinically. Pumps were essential	1, 2, 4, 5, 6, 7

### 3.2 The practical impact of infant hospitalization on the parent

Infant hospitalization impacts parents in a very practical way. Eight of the 11 studies found various practical problems, ranging from issues of logistics to practical breastfeeding problems. For example, the way in which the infant was fed breastmilk often had to change due to separation or necessary adaptations due to the illness/disorder. Many mothers had to start expressing milk or work to maintain supply when under normal circumstances they may have been able to directly feed (Lambert & Watters, 1998). This can add in a layer of complication in finding the time to express, store and deliver the milk.

Expressing milk rather than directly breastfeeding is also not necessarily straightforward. It is associated with a higher risk of blocked ducts, mastitis, engorgement (Kvist, 2010) and ultimately low milk supply if the child is not effectively removing milk (Morton et al., 2013). Indeed, Barros da Silva et al. (2019) found that breast‐related problems such as engorgement, nipple trauma and difficulty with milk expression were common, and several studies highlighted the issue of perceived low milk supply, sometimes leading to formula supplementation or breastfeeding cessation (Madhoun et al., 2019; Rendón‐Macías et al., 2002; Ryan et al., 2013). Mothers were therefore left dealing with the complications of this on top of the practical complications of ensuring their infant received the milk. However, mothers feel a desire to persevere with expressing, describing it as a labour of love (Banta‐Wright et al., 2015).

There were other more logistical problems as well that were unrelated to breastfeeding physiology. For example, Heilbronner et al. (2017) noted the challenge of staying overnight in hospital, whereas Rendón‐Macías et al. (2002) identified the difficulty when infants were hospitalized far from home, especially when it came to juggling paid work.

### 3.3 The impact of infant hospitalization on the parent: Psychological

Nine of the studies explored data relating to the parental psychological aspects of breastfeeding their hospitalized child. These could be both negative and positive. For example, from a negative perspective, Duhn and Burke (1998) and Lewis and Kritzinger (2004) found that exhaustion, overwhelm, disappointment, frustration, inadequacy, loneliness and sadness were common. Likewise, Madhoun et al. (2019) described high levels of anxiety and depression in their sample, with over half the mothers suffering with anxiety and one third struggling with postnatal depression.

However, Lambert and Watters (1998) found some positive aspects relating to breastfeeding a child with a chronic condition. Some of the perceived benefits of breastfeeding included a greater sense of calm, decreased stress, an opportunity for relaxation and an increased sense of self‐efficacy.

### 3.4 The impact of infant acute critical illness or instability affecting infant ability to breastfeed

Eight of the studies commented on infant instability or severity of illness as a factor affecting their ability to feed easily. Of these studies, one was a study of children with acute bronchiolitis in otherwise healthy children, and the others all described experiences of children with congenital conditions.

Several studies exploring congenital heart defect found infant critical illness or instability to be a barrier to breastfeeding. Duhn and Burke (1998) found that infants with heart defects were likely to struggle to feed and be too fatigued to feed, and surgery and ventilation were cited as factors affecting an infant's ability to breastfeed. Barbas and Kelleher (2004) found that infants were more likely to be breastfed postoperatively if they had attempted breastfeeding before their surgery. Meanwhile, Lambert and Watters (1998) and Rendón‐Macías et al. (2002) cited preoperative fasting as a significant barrier to breastfeeding.

Problems were also noted among infants with Down syndrome and Rubinstein–Taybi syndrome, both of which are often associated with co‐morbidities (Moe et al., 1998). Barros da Silva et al. (2019) found that infants with Down syndrome were more likely to have problems with breastfeeding if they also had a cardiac defect, low tone and poor suck.

The only study to explore an acute illness was Heilbronner et al. (2017). It appeared that admission to hospital for acute bronchiolitis was associated with reduced exclusivity or cessation of breastfeeding, but the reasons for this were multifactorial and mostly unrelated to illness severity.

### 3.5 The impact of infant chronic condition on their ability to effectively breastfeed

All but one paper (Heilbronner et al., 2017) discussed the impact of certain symptoms of the infant's condition on their ability to breastfeed or the duration of breastmilk provision. Infant conditions generally caused hypotonia, somnolence and poor weight gain in a cluster or drove a need for feeding adaptations such as frequent short feeds and strategies to increase calories to manage fatigue and growth problems. Banta‐Wright et al. (2015) was the exception to the previously mentioned adaptations to feeding. In their study, they found that mothers had to be constantly flexible, in response to their child's phenylalanine (Phe) levels, not because of their infant's tone or somnolence, but because of their underlying metabolic condition.

Most of the studies exploring the challenges of breastfeeding infants with cardiac defects had many findings in common. Increasing and measuring milk consumption was a common theme. Barbas and Kelleher (2004) focused mainly on the need for more calories, whereas Lambert and Watters (1998) and Duhn and Burke (1998) found that many mothers needed to provide small frequent feeds to manage poor weight gain. Meanwhile, a poor suck and subsequent low weight gain were identified by Rendón‐Macías et al. (2002) particularly among children with congenital anomalies. Finally, Ryan et al. (2013) identified the difficulty of needing to know feed volumes to manage an infant's clinical condition.

Further studies focused on somnolence and hypotonia, including Moe et al. (1998) and Barros da Silva et al. (2019). Lewis and Kritzinger (2004) cited hypotonia, poor and weak suckling, uncoordinated suck–swallow–breathe reflex sequences, macroglossia, small intra‐oral space and difficulties achieving a comfortable and effective position due to low tone.

Finally, Madhoun et al. (2019) studied 150 children with cleft lip and palate. They found that a cleft palate was associated with more problems breastfeeding, irrespective of whether there was also a cleft lip. The infants with only a cleft of the lip had the highest rates of breastfeeding. In this study, most of the mothers expressed their milk either long term or prior to surgical correction of the cleft. The type of cleft made no difference to the duration of expressing—only direct breastfeeding.

### 3.6 The availability of specialized lactation support in the hospital paediatric setting

A total of nine papers commented that lack of specialist lactation support made breastfeeding harder. For example, Banta‐Wright et al. (2015) found that although there were designated International Board Certified Lactation Consultants (IBCLCs) in the neonatal unit, they were not employed in paediatrics. One study participant telephoned every IBCLC she knew to ask for information and managed through this convoluted route to get the help she needed. Meanwhile, in the Barros da Silva et al. (2019) study with mothers of infants with Down syndrome in Portugal, mothers mainly expressed dissatisfaction with the support they received, mostly from nurses. Most reported that there was very little support or information, and they also had little encouragement to breastfeed. Likewise, in Ryan et al.'s (2013) study of infants with various chronic illnesses, mothers raised the issue of a lack of reliable, sensitive and accessible breastfeeding support. This was echoed in the Lambert and Watters (1998) study where physician support was rated as least helpful. However, when mothers did have access to an IBCLC, they found it useful.

Details of who is providing specialist support are often unclear in published research. For example, Heilbronner et al. (2017) studied 84 infants admitted to a hospital without baby friendly accredited status in France with acute bronchiolitis. A total of 51% of the mothers either stopped or reduced the exclusivity of breastfeeding. In this study, each ward had several breastfeeding ‘experts’ among the doctors and nurses, but no specific lactation support service. It is unclear how the definition of expert is made.

Some mothers turn to other sources for information and support instead. For example, Lewis and Kritzinger (2004) found peer support from another mother who had breastfed a child with Down syndrome particularly helpful, although this was not a standardized service. Meanwhile, Madhoun et al. (2019) and Moe et al. (1998) found that mothers accessed online support groups and organized breastfeeding support groups were also identified.

When high‐quality support was provided, it had a positive impact. For example, Barbas and Kelleher (2004) studied 68 infants with congenital heart disease (CHD) in the United States, where 6 years previously they had established a designated lactation support programme led by a full‐time IBCLC. The IBCLC also had extensive paediatric nursing experience and went on to establish a programme of education for all staff. This intervention led to an increase in breastfeeding rates from 14% to 47% in that time. In this study, mothers cited the IBCLCs as very supportive.

### 3.7 The support, training and attitudes of healthcare professionals

In general, most of the studies highlighted inadequate support. Many of the healthcare staff were acknowledged as caring, but most parents did not get the breastfeeding support they needed. Some staff were perceived as ambivalent about the importance of breastmilk (Barbas & Kelleher, 2004) or unaccepting of breastfeeding (Barros da Silva et al., 2019). Duhn and Burke (1998) highlighted negative language used by health professionals, for example, referring to infants being ‘starving’. Other studies identified a lack of useful support. For example, Heilbronner et al. (2017) attributed breastfeeding cessation or formula use to unhelpful advice, whereas Lambert and Watters (1998) reported that women rated paediatric staff knowledge as poor.

A lack of training and skills was identified by a number of studies as central to the lack of support (Lambert & Watters, 1998; Madhoun et al., 2019). Moe et al. (1998) found that parents perceived physicians to be theoretically supportive of breastfeeding, but without adequate training to be able to provide support for breastfeeding challenges. Rendón‐Macías et al. (2002) found that advice to supplement or stop breastfeeding by a medical professional was prevalent. In the Ryan et al. (2013) study, mothers highlighted various gaps in knowledge. Banta‐Wright et al. (2015) found that parents had to be creative, finding their own breastfeeding support or utilizing peer and family support.

### 3.8 The necessity and availability of specialized equipment or resources

Finally, all but one study (Rendón‐Macías et al., 2002) discussed breastfeeding equipment. Although nearly all the parents required specialized equipment, access to such products was not always universal and support was weak. The participants in the Duhn and Burke (1998), Lewis and Kritzinger (2004), Banta‐Wright et al. (2015) and Barros da Silva et al. (2019) studies variously used breast pumps, bottles, nasogastric tube (NGT), gastrostomy tube (GT) and syringes but did not in general find adequate support to help them with their breastfeeding journeys. Lambert and Watters (1998) identified lack of privacy, lack of access to pumps and inconsistent advice as barriers. Heilbronner et al. (2017) noted that many parents complained of breast pump shortage and that pumping was difficult, creating a significant barrier to expressing.

In the Madhoun et al. (2019) study, there is specific mention of six different specialty feeding bottles, as well as NGT and GT. Some of the mothers were disappointed that the hospital staff did not know how to help the parents use the equipment. Finally, Ryan et al. (2013) found that mothers were reliant on practical aids such as a specialized bra that enabled hands‐free pumping. Three mothers who used NGT felt that they were at times overused instead of attempting breastfeeding.

## 4 DISCUSSION

This study aimed to establish the existing body of literature relating to the needs and challenges of breast/chestfeeding medically complex infants and children in the paediatric setting and identify gaps in healthcare provision that may serve as barriers to maintaining lactation and facilitating breastfeeding in this population. There is a paucity of original research in this area, with almost none relating to older babies and toddlers. Much more work is needed in order to make specific recommendations for changes in practice. The existing research has focused on specific conditions. This makes generalization much harder and therefore less clinically implementable for most paediatric wards admitting children with a range of illnesses. Without extensive background knowledge of how multiple conditions may impact breastfeeding in unique ways, this may mean that unless there is a specific guideline for every condition, health professionals may not be able to apply tools that work for children with disparate conditions, even though the challenges may be similar. Arguably, what would be more useful and user‐friendly for practitioners, especially those without extensive lactation training, would be a guideline that provides practical tools and suggestions for challenges by theme—such as low tone, somnolence or higher caloric need, rather than by condition.

However, despite the limited research, seven themes emerged from the available literature, relating to practical and psychological challenges for parents, difficulties associated with clinical instability and physical condition of infants, specialized lactation support, healthcare professional skills and attitudes and specialized equipment and techniques.

There are numerous practical problems relating to the hospitalization of infants and children. These may be practical breastfeeding problems caused by disruption to the normal process of responsive feeding, for example, blocked ducts and low milk supply, as well as logistical problems. In the papers in this study, the main non‐lactation‐related practical problems were challenges relating to being resident in hospital away from home and needing to balance paid work with caring for their child. Although the papers in this study only briefly alluded to financial strain, other literature relating more generally to the impact of hospitalization on families cites the financial burden of transport, parking costs and food and other items that usually have to be bought from the on‐site hospital shop (Mooney‐Doyle et al., 2018). The papers in this study had limited numbers of low‐SES families, which may be significant as these problems will have the greatest negative impact on vulnerable and low‐income families (Beck et al., 2017; Thomson et al., 2016).

Most parents in this study cited some level of psychological distress surrounding their child's admission to hospital, although some specifically described the positive benefits that breastfeeding brought to the experience. The parents cited exhaustion, stress, anxiety and depression frequently. The psychological aspects were not all negative however, with many of the mothers describing breastfeeding as something that made them part of the solution, and one mother stated that she felt breastfeeding helped to re‐establish trust with her toddler after their surgery. Essentially, breastfeeding was hard work, but the parents were motivated to continue despite the challenges. The psychological challenges relating to breastfeeding may on the one hand negatively impact a parent's confidence and experience of feeding and caring for their child, but breastfeeding also provides an opportunity to empower parents to feel included in their child's care. Supporting parents to be able to overcome a challenge rather than feel defeated by it may lead to a greater sense of self‐efficacy. Throughout many of the papers, there was a sense that the parents managed to persevere with breastfeeding in spite of their experience within the paediatric setting, rather than because of it.

There were also infant‐related feeding challenges, distinctly different from the practical challenges of maintaining healthy lactation in the parent. Not all congenital conditions affect a child's immediate physiological stability, such as cleft palate or Down syndrome. However, even when a child is initially stable, their condition can change, or corrective surgery can make them more unstable.

Some conditions necessitate specific breastfeeding adaptations due to the infant's condition, and not their medical instability. These may be related to positioning for breastfeeding, fat or calorie content, specialized techniques or frequency of feeding. Effective breastfeeding involves both the infant and parent. The infant has to be able to use their tongue, lips, jaw and cheeks to stabilize the breast in their intra‐oral palate, create negative pressure and be able to safely suckle and swallow while also coordinating breathing (Genna, 2013). However, for ongoing successful lactation, milk must be removed from the breast/chest according to the infant's individual metabolic and caloric need. The infant will need to be positioned sustainably for breastfeeding in a way that supports a safe suck–swallow–breathe sequence.

Some children are born with conditions that require breastfeeding modification. For example, infants with chylothorax cannot receive breastmilk unless it has been separated in a centrifuge to remove the fat (Davis & Spatz, 2019), and infants with phenylketonuria (PKU) cannot breastfeed exclusively because although breastmilk contains less phenylalanine than formula, these infants usually need specialized Phe‐free formula to a greater or lesser extent depending on their Phe levels—which must be monitored closely. Conversely, infants with hypotonia may not only tire easily but are also more difficult to hold and position, and they may not be able to effectively create a seal at the breast. Supporting a mother–baby dyad in these specialist cases is more difficult and requires specialist knowledge compared with supporting healthy breastfeeding infants.

In many clinical settings, such as maternity or neonatal units, specialist lactation support is a clearly defined sub‐specialty. This type of support involves more than simple breastfeeding management in uncomplicated situations and requires the ability to be able to assess and treat complications, at a level far higher than standard breastfeeding training. Globally, the IBCLC credential is the recognized leading qualification in breastfeeding support, and IBCLCs have the most comprehensive and robust skill sets (Chetwynd et al., 2019).

However, the number of IBCLCs globally varies, as does the scope of practice. In the United States, IBCLCs are often part of the wider healthcare team, serving neonatal and obstetric departments (Haase et al., 2019). Conversely, in other countries, such as the United Kingdom, IBCLCs usually only work in the hospital setting if they are also a health professional. Although their additional skills enable them to effectively carry out their role, the credential itself is often incidental, and not formally part of the person specification. Other staff may not always have specialist breastfeeding knowledge and skills, meaning parental experience can differ depending on who they encounter (Holaday et al., 1999; Dykes, 2006; McLaughlin et al., 2011). Additionally, lactation support is often limited to maternity and neonatal care units, meaning it is often not routinely present on paediatric units.

Alongside specialist services, we know that breastfeeding is best facilitated when all health professionals looking after a mother recognize its value and have the skills to support her or signpost for more specialist support if needed (McFadden et al., 2017; Thomas, 2020). However, although UNICEF Baby Friendly standards support and protect breastfeeding on the neonatal and maternity wards, these do not currently extend into paediatrics (Carney & Bruce, 2011). Therefore, there are no standardized, mandatory training programmes for paediatric nurses, physicians and allied health professionals such as dieticians, speech and language therapists, physiotherapists and occupational therapists—all of whom are likely to work with medically complex children. The World Breastfeeding Trends Initiative (WBTi, 2020) identified many risk factors among the training curriculums of health professionals, noting that there are many gaps (Gupta et al., 2019).

The support, training and attitudes of health professionals are considered as a separate theme, as some units and hospitals in the review had designated lactation support that was considered alongside medical treatment. Other units and hospitals had no such identified service, and therefore, any lactation support was provided by the medical team—who may or may not have the required skills and training to offer support.

When direct, exclusive, responsive breastfeeding is not possible, extra feeding equipment will be needed, both for parents and for infants. For example, parents will need to maintain their milk supply with a breast pump (Marasco, 2008) —either a hospital‐grade double electric pump, single electric pump or manual breast pump, together with hand expressing and breast massage (Geddes et al., 2013; Morton et al., 2009; Morton et al., 2012; Morton, 2014; Witt et al., 2016; Strauch et al., 2019). Different approaches work best for individual mothers (Meier et al., 2016). Specialized bottles and teats (e.g. squeeze bottles and one‐way valves), cups, spoons, syringes, NGT, nipple shields, palatal prostheses, at‐breast supplementers and GT may also be needed (Rosenberg et al., 2008; Boyce et al., 2019; Rudra et al., 2016). A thin silicone nipple shield may increase the effectiveness of milk transfer for infants unable to achieve good intra‐oral pressure at the breast (Meier et al., 2017). Parents will likely need further education around using these products and maximizing milk supply.

Very few of the studies specifically studied the use, education or availability of equipment. There are many aspects of using specialized equipment that are missing, such as the possibility of expressing milk at the infant's bedside, how to optimize milk production in difficult circumstances and utilize specialist equipment and specific techniques for positioning infants with low tone, fatigue or orofacial anomalies.

### 4.1 Limitations of this review

A major limitation of this review is that it was conducted by a single reviewer. This was unavoidable as it forms part of a PhD. The process was made more rigorous by a second reviewer checking the criteria used and being involved in the development of the review. This systematic review is also small, so all co‐authors became familiar with the studies analysed.

No study explored the impact of illness in a general sense on breastfeeding. There is a paucity of research related to infant acute illness and serious conditions that do not specifically affect the head, mouth, palate or face.

The available studies have all explored the relationship between illness/disability and breastfeeding outcome in a disease‐specific way, without drawing out more general themes. Becasuse all infant and child conditions will affect breastfeeding differently, with so few conditions studied it is hard to know whether some aspects of infant feeding difficulty have not yet been identified. The data are therefore not necessarily generalizable.

Additionally, most paediatric wards admit children with a range of diseases and illnesses. It is perhaps more user‐friendly for health professionals who may not have specific expertise in infant feeding to have general guidelines for supporting breastfed children and families in the paediatric setting rather than expecting medical and nursing staff to search for disease‐specific protocols.

The quality, time span and global variation reported in the research included in this review limit the ability to form solid conclusions. Most of the studies were small, and none of them fully addressed all of the potential confounding variables. Many of the studies were samples of committed mothers. It is unknown how breastfeeding outcomes would differ among less motivated samples.

The studies included also tended to have limited racial diversity and SES among the included participants. This may be representative of the ongoing higher prevalence of breastfeeding in high‐SES groups and among predominantly White, married, heterosexual, women with higher levels of education (Bartick et al., 2017). Again, this limits the generalizability of the findings to the wider population.

In addition, the studies came from different parts of the world where healthcare systems are disparate. Half of the studies were conducted in the United States or Canada. This is potentially problematic in terms of exploring healthcare‐based lactation support, as the provision of healthcare and IBCLC‐led expertise is different around the world.

Furthermore, the studies available span 20 years, in which time breastfeeding support and training has evolved and breastfeeding rates have generally improved. The BFI has expanded significantly since its inception in 1991, with more protection for breastfeeding thanks to initiatives such as the World Breastfeeding Trends Initiative and increased awareness of the WHO International Code (WHO, 1981).

Finally, it is likely that other studies exist that explore the challenges of breastfeeding sick children, which did not use terms specific enough to have been identified. However, removing the search terms relating to paediatrics would conversely have limited the sensitivity of the search.

## 5 CONCLUSIONS

There is much we know about breastfeeding in terms of risk reduction of various illnesses (Victora et al., 2016), yet we know far less about what it is like to breastfeed a medically complex child. Although breastfeeding reduces the risk of many conditions, it does not eliminate risk. In the important work of continuing to promote of breastfeeding in general, we must not forget the children who are unwell despite having been breastfed.

There are already recommendations for certain conditions, for example, cancer (Carney, 2013), cystic fibrosis (Luder et al., 1990) and insulin‐dependent diabetes (Miller et al., 2017). For this review question, the only conditions explored were Down syndrome, CHD, cleft palate, PKU and Rubinstein–Taybi syndrome, but many suggestions for specific conditions could be adapted and summarized in order to increase generalizability to other conditions that have not yet been studied.

This review has identified seven themes relating to why breastfeeding medically complex children is more challenging. Breastfeeding difficulties may be parent oriented or child oriented, relating to specialist lactation support, healthcare professional support and training and necessary practical equipment. This work could impact the scope and reach of the BFI and potentially evidence the need for its extension in the paediatric setting, with specific training and audit for staff working in this area.

Future research should address these identified barriers in order to improve the experience of families breastfeeding through challenging circumstances. There also needs to be a concerted effort to address the training needs of healthcare teams caring for sick infants and children in the hospital setting. Future training needs to be differentiated from breastfeeding education aimed at the initiation and management of basic breastfeeding problems in healthy parent–child pairs. Parents and children resident in hospital are likely to have different needs and require additional or specialist support that is not currently addressed in mainstream breastfeeding education. Exactly what these needs and differences are, and what the training programme would look like, are important research questions that should be addressed in further work in order to optimize the health outcomes for medically complex children.

## CONFLICTS OF INTEREST

The authors declare that they have no conflicts of interest.

## CONTRIBUTIONS

LH was responsible for study conception, selecting and reviewing all articles, draft manuscript completion and critical revisions; JL was responsible for study conception and critical revisions; AB was responsible for study conception, draft manuscript support and critical revisions.

## Data Availability

Data sharing not applicable to this article as no datasets were generated or analysed during the current study.

## APPENDIX A Summary of reviewed articles

**Table:**

Article	Country	Pub year	Objective	Design	Main findings	Pop'n type	Number in sample	Potential confounding factors?	Variables controlled for/commented on?
Banta‐Wright et al.	USA/Canada	2015	Describe meaning and purpose of BF to mothers	Qualitative descriptive	Diagnosis required a re‐commitment to BF, including learning about child's condition BF was meaningful, maintaining closeness and connection, but required some adaptation—being flexible about how much/little BF, as well as maintaining lactation Mothers required support. Mostly, this came from peers. Most cited a lack of clear written info. LCs helped but had to be found Final theme was that although BF infants with PKU was hard work, it was worth it. BF gave them a way to see their infants as normal	Mothers of babies with PKU	10	SES, intention to breastfeed, education status, previous breastfeeding experience, prior knowledge of PKU, support from partner, support from healthcare professionals, information about PKU, NICU/PICU admission, gestational age, availability of breast pump, distance from hospital or access to regular domiciliary nursing for blood tests, BFI‐accredited hospital, parental mental health condition	SES, partner availability, education status, previous children with PKU
Barbas and Kelleher.	USA	2004	Describe BF outcomes among mother–infant pairs in a CHD context	Retrospective survey	Breastfeeding provided mothers with an opportunity to feel more involved in their infant's care, as well as higher sense of self‐efficacy, making a ‘difficult experience bearable’ Many mothers found nursing and medical staff unsupportive—the importance of breastmilk was not acknowledged by many, and many of the staff were felt to imply that formula was better and less ‘icky’. There were some notable exceptions from supportive staff members The most common positive comment was about the availability of pumps	Mothers of babies with CHD	68	SES, intention to breastfeed, education status, previous breastfeeding experience, antenatal breastfeeding education, access to specialist breastfeeding support, NICU/PICU admission, gestational age, breast pumps, support from healthcare professionals, infant caloric need/growth rate, BFI‐accredited hospital, parental mental health condition	Maternal age, level of education, marital status, number of children, classification of CHD, prenatal preparation for CHD, availability of specialist breastfeeding support
Barros da Silva et al.	Portugal	2019	Explore experiences of mothers breastfeeding children with Down syndrome	Qualitative, semi‐structured interview	Mothers expressed dissatisfaction with healthcare practitioners regarding their support and knowledge of breastfeeding children with Down syndrome. Mothers persevered due to their commitment to breastfeeding, in spite of a lack of support and the challenges of feeding babies with low tone, sucking problems or cardiac anomalies	Mothers of children with Down syndrome	10	SES, education status, intention to breastfeed, previous breastfeeding experience, antenatal education, prenatal diagnosis of Down syndrome, co‐morbidities (such as CHD), support from health professionals, infant stability, NICU/PICU admission, gestational age, availability of psychological support, BFI‐accredited hospital, parental mental health condition	Confounding variables not commented on
Duhn and Burke	Canada	1998	What are the key issues experienced by mothers feeding their babies with CHD	Qualitative interviews using a grounded theory approach (unpublished)	There was a theme of acknowledging that the feeding and mothering process was different. This involved grief and shock Mothers described the hard work of feeding, and incorporating this into everyday life was difficult, stressful, time consuming and anxiety provoking Mothers began to develop feelings of control after reframing their experience, persisting with feeding even though it was hard and choosing to remain positive There were recurring experiences of loss, fear of their baby dying and an emotional battle between feeling close through feeding, yet needing to create a protective distance due to a feeling of threatened survival All mothers stopped breastfeeding within 4–6 weeks, and though some continued to pump, nobody resumed breastfeeding after initially stopping	Mothers of infants with CHD	7	SES, education status, previous breastfeeding experience, BFI‐accredited hospital, parental mental health condition	
Heilbronner et al.	France	2017	Evaluate breastfeeding disruption during hospital admission for bronchiolitis	Cross‐sectional study	Forty‐three per cent of mothers stated that hospitalization modified their breastfeeding experience. Several either stopped, switched to partial breastfeeding or reduced breastfeeding. Lack of support by healthcare staff as well as medical advice was the most commonly cited reason for this	Breastfeeding mothers of children hospitalized with bronchiolitis	84	Child age, prior difficulty with breastfeeding, social support, SES, intention to breastfeed, PICU admission, ventilation, respiratory support, tube feeding, whether parent was able to be resident overnight, nursing ratios, level of nursing knowledge in breastfeeding, problems with recall, availability of breast pump, BFI‐accredited hospital, parental mental health condition	Age of child, length of stay, severity of illness, length of PICU stay, length of ventilation, nutritional support, growth rate prior to hospitalization. SES only available for 54/84 patients
Lambert and Watters	Canada	1998	Share insights about the experiences of parents of CHD infants	Descriptive survey	Some parallels noted between maternal reported benefits of breastfeeding a sick child with those reported by mothers of preterm infants. Most of the mothers were not encouraged to breastfeed, and many healthcare professionals expressed inaccurate views of breastfeeding in the context of physiological instability. There were also barriers put up by healthcare professionals due to concerns over the difficulty with measuring volumes when babies are breastfed	Mothers of children with CHD	12	SES, education status, intention to breastfeed, previous breastfeeding experience, degree if infant stability, NICU/PICU/CICU admission, gestational age, antenatal education, prenatal diagnosis of CHD, peer support, availability of specialized lactation support, support from healthcare staff, BFI‐accredited hospital, parental mental health condition	Maternal age, education status, nature of cardiac anomaly, personal breastfeeding goals, whether information provided by medical professionals was broadly positive, negative or absent
Lewis and Kritzinger	South Africa	2004	Describe the experiences of feeding children with Down syndrome	Descriptive survey	There were a number of feeding challenges due to low tone, heart defects, infant exhaustion, problems with sucking and safe swallowing Mothers experienced a range of emotions, such as shock, concern, stress, disappointment and frustration Mothers identified that they required support, choices and skilled guidance from professionals, as well as peer support to achieve their feeding goals They also valued encouragement to continue breastfeeding	Mothers of children with Down syndrome	20	SES, education status, intention to breastfeed, previous breastfeeding experience, comorbid condition, prenatal diagnosis of Down syndrome, NICU/PICU admission, gestational age, nutritional/feeding support, BFI‐accredited hospital, parental mental health condition	Maternal age, but not level of education, partner status or antenatal education Many infant variables accounted for. Apart from NICU/PICU admission—though questions were asked about whether baby was ventilated
Madhoun et al.	USA	2019	To examine trends in breastmilk provision and characterize barriers and supports to maintaining breastfeeding or breastmilk feeding	Online retrospective cross‐sectional study of parents of cleft babies	Breastfeeding duration was dependent on cleft type. CL‐only babies were more successful at breastfeeding Many mothers in the study pumped, and pumping duration was not affected by cleft type Lactation consultants were the most common source of support, but were not ‘required’ members of the cleft team. Feeding duration was also improved by peer support Many mothers described anxiety and/or depression	Mothers of babies with cleft lip/palate	150 (69 BF mothers)	SES, education status, previous breastfeeding experience, intention to breastfeed, partner support, lactation support, medical professional support, access to breast pump, NICU/PICU admission, cleft lip and/or palate, co‐morbidities, BFI‐accredited hospital, parental mental health condition	SES, maternal age, marital status, education status, intention to breastfeed, age of infant, cleft type, co‐morbidities, maternal depression, NICU admission
Moe et al.	USA/Canada	1998	Determine what support is useful in establishing and maintaining lactation in infants with Rubenstein‐Taybi syndrome	Retrospective survey	Even with very little support, babies with RTS demonstrated ability to be able to breastfeed successfully. However, mothers reported that many health professionals were discouraging, and there were not enough lactation consultants to support them with specialist feeding techniques to facilitate effective feeding. Support more often came from booklets, family members and their prior experience of breastfeeding another child. The research suggests ways in which the techniques described in this study may be of help to babies with other conditions that involve low tone—such as Down syndrome	Parents of children with Rubenstein‐Taybi syndrome	194	SES, education status, intention to breastfeed, previous breastfeeding experience, NICU/PICU admission, co‐morbidities, lactation and healthcare professional support, BFI‐accredited hospital, parental mental health condition	No data on SES variables, parental age, partner status, prenatal preparation. Data were provided about breastfeeding duration, reasons for cessation, quality and prevalence of support provided and by whom
Rendón‐Macías et al.	Mexico	2002	Determine frequency of breastfeeding, and identify factors associated with initiation and cessation among parents of children with congenital anomalies	Descriptive cohort study	Infants with congenital malformations are less likely to BF. Mothers cited many reasons, including medical advice, separation and infant disease—especially GI disease	Mothers of babies with congenital anomalies	120	SES, education status, previous breastfeeding experience, BFI‐accredited hospital, intention to breastfeed, infant condition, co‐morbidities, NICU/PICU admission, breast pump availability, parental mental health condition	No data relating to SES, partner status, education level, provided Data about parental age, employment, intention to breastfeed, antenatal education, initial infant feeding pattern and infant condition are provided. No breast pumps available at this facility, and no rooming in facilities—though parents are permitted to stay (unclear how this is facilitated). Unclear differentiation between exclusively and partially breastfed infants when calculating duration of breastfeeding
Ryan et al.	UK	2013	Explore the experience of breastfeeding a baby with chronic illness or disability	Narrative interviews	Chronic illness or disability causes disruption to the breastfeeding relationship. In this study, mothers' sense of self‐efficacy was closely tied to their ability to breastfeed and thus was an important part of emotional adjustment to chronic illness or disability	Mothers of children with Down syndrome, cleft and CHD	5	SES, education status, previous breastfeeding experience, intention to breastfeed, infant condition, NICU/PICU admission, co‐morbidities, BFI‐accredited hospital, parental mental health condition	Parental age, marital status, employment status, ethnicity was collected. Data about infant illness or disability were also provided

## APPENDIX B Checklist for cross‐sectional study adapted from CASP (2018) cohort study

**Table:**

CASP criteria	Met the criteria? (yes, cannot tell, no)
Q1: Did the study address a clearly focused issue?	
Q2: Was the sample recruited in an acceptable way?	
Q3: Was the outcome accurately measured to minimize bias?	
Q4a: Have the authors identified all important confounding factors?	
Q4b: Have the authors taken account of confounding factors in their design and analysis?	
Q5: Was the follow‐up of subjects complete enough?	
Q6: What are the results of this study?	
Q7: Do you believe the results?	
Q8: Can the results be applied to other populations?	
Q9: Do the results of this study fit with other available evidence?	
Q10: What are the implications of this study for practice?	
